# Early-Stage Renal Cell Carcinoma: Who Needs Adjuvant Therapy?

**DOI:** 10.3390/biomedicines13030543

**Published:** 2025-02-21

**Authors:** Andreea Ioana Parosanu, Cornelia Nititpir, Ioana Miruna Stanciu, Catalin Baston

**Affiliations:** 1 Faculty of Medicine, Carol Davila University of Medicine and Pharmacy, 8 Sanitary Heroes Boulevard, 050474 Bucharest, Romania; cornelia.nitipir@umfcd.ro (C.N.); ioana-miruna.stanciu@drd.umfcd.ro (I.M.S.); catalin.baston@umfcd.ro (C.B.); 2 Department of Oncology, Elias University Emergency Hospital, 011461 Bucharest, Romania; 3 Department of Urology, Fundeni Clinical Institute, 022328 Bucharest, Romania

**Keywords:** renal cell carcinoma, adjuvant therapy, risk factors, clinical trials

## Abstract

Surgery is the oldest modality of kidney cancer therapy and is usually the first step in the treatment process. To improve surgical outcomes, adjuvant therapy is frequently administered to eliminate residual tumors and reduce the risk of recurrence and metastasis. However, not all patients require adjuvant treatment. The decision regarding whether to treat or not to treat renal cell carcinoma patients depends on the risk of recurrence, including tumor stage and histology, and clinical, biological, and personal risk factors. This article will address the challenges of treating renal cell carcinoma patients with adjuvant therapy and review the current evidence and ongoing clinical trials.

## 1. Introduction

In 2022, more than 430,000 renal cell carcinoma (RCC) cases were reported worldwide, with over 2600 new cases recorded in Romania [1]. More than 70% of patients are diagnosed with localized non-metastatic RCC, specifically stages I, II, and III [2].

Several management options exist for clinically localized renal masses where RCC is suspected. Active surveillance is a viable initial management strategy for clinical stage T1 renal tumors (limited to the kidney and measuring less than 7 cm) [3]. Additionally, ablative techniques, such as thermal ablation or stereotactic body radiotherapy have become accepted treatments for these small masses. This approach is intended for RCC patients who are not candidates for surgery, and in such cases, a tumor biopsy should be performed before or during the ablation process [4]. Traditional treatments for localized RCC include open, laparoscopic, or robotic radical and partial nephrectomy [5].

Regional lymph node dissection is the most accurate and reliable method for disease staging. Although optional, it should be considered for patients with resectable lymph nodes identified on preoperative computed tomography scans or magnetic resonance imaging [6,7].

Approximately 20% of patients with RCC experience a recurrence following nephrectomy [8,9]. Consequently, standard surgical therapy alone is often inadequate for achieving long-term tumor control. To improve surgical outcomes, adjuvant therapy is frequently administered after primary treatments to eliminate any residual tumors and reduce the risk of recurrence and metastasis [10].

However, not all patients with RCC require adjuvant therapy. Resorting to it depends on several clinicopathological risk factors and whether the benefits of adjuvant therapy outweigh its adverse reactions and treatment toxicities.

In this article, we discuss patients at risk of recurrence after kidney cancer resection and strategies to effectively identify those who would benefit most from adjuvant therapy. Furthermore, we review the available evidence on adjuvant therapy for RCC and explore potential future strategies.

## 2. Risk Stratification in RCC: Who Is at the Highest Risk of Recurrence?

Identifying patients at the highest risk of disease progression and mortality is critical for making informed decisions regarding adjuvant therapy. Retrospective real-world data clearly show that the morphological types of RCC and tumor stages play a vital role in assessing recurrence risk and prognosis [11]. Although pathological staging is one of the most crucial factors in predicting cancer progression and death after surgery, relying solely thereon can fail to capture the full spectrum of an individual patient’s risk [12]. Therefore, additional clinicopathological factors are needed to better predict oncological outcomes. These factors include tumor size, histology, grade, tumor necrosis, nodal involvement, rhabdoid or sarcomatoid features, and specific symptoms at diagnosis [13]. Understanding these elements is essential for effective risk assessment and optimizing patient care.

### 2.1. Tumor Stage

Similar to most cancers, *RCC* stage and *grade* are linked to the tumor’s biological potential [11]. Consequently, the risk of metastasis and recurrence increases steadily with tumor size and grade.

**Figure 1:**
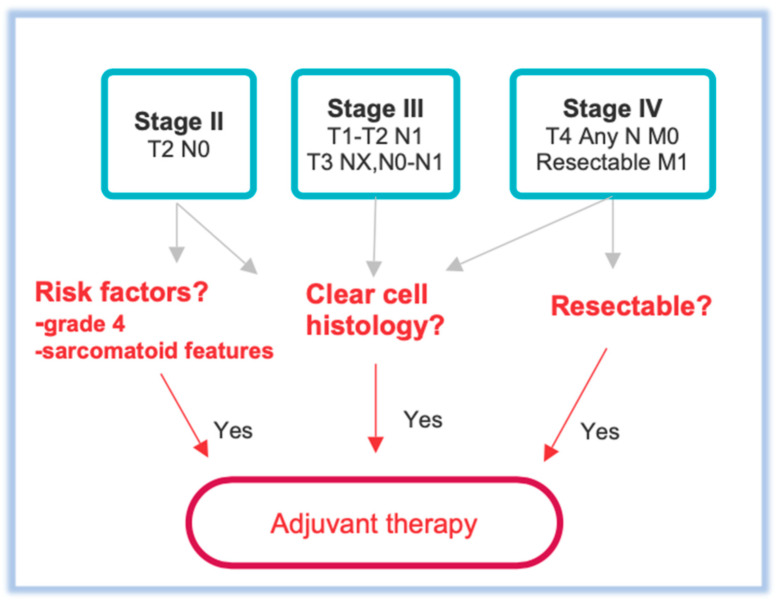
Indications for adjuvant treatment based on tumor stage.

According to National Comprehensive Cancer Network (NCCN) guidelines, surveillance is indicated for patients with stage I, while adjuvant treatment is recommended for stages II or III after surgery (Figure 1) [14]. There is a considerable difference in the anatomy of spreading tumor cells and their prognosis between stages II and III. Stage II RCCs have significantly better 5-year survival rates (83.4%) compared to stage III (66.0%) [15,16].

Stage III indicates that the cancer has become more advanced and has spread into the fatty tissue surrounding the kidney. It may also involve the renal vein or even the vena cava and could spread to the lymph nodes. Adjuvant therapy is essential in such situations [17,18].

Stage II means the cancer is confined to the kidney, is larger than 7 cm, and has not spread to the lymph nodes or other organs. Adjuvant therapy is indicated not for all stage II RCCS but only for those with high-risk features of recurrence, which include grade IV tumors with clear cell histology or sarcomatoid characteristics [11].

Adjuvant treatment is also a viable option for patients with resectable stage IV tumors. Some patients with locally advanced tumors that have spread beyond the Gerota fascia or involve the ipsilateral adrenal gland, but without distant metastases, may be considered for this treatment after undergoing nephrectomy, especially if they have clear cell histology. Further, in patients with clear cell histology and stage IV RCC who have undergone a complete metastasectomy, adjuvant therapy may also be considered as a treatment option [11,19].

### 2.2. Tumor Grade

Pathological staging plays a vital role in evaluating the risk of cancer progression and potential mortality following surgery. Following World Health Organization (WHO) guidelines, any RCC demonstrating sarcomatoid dedifferentiation is classified as a grade IV lesion by the WHO International Society of Urological Pathology, highlighting the importance of accurate classification in informing treatment decisions [20,21]. However, relying solely on this staging approach can result in a limited understanding of a patient’s overall risks. For better patient outcomes, a more thorough evaluation strategy is imperative.

### 2.3. Tumor Histology

According to the 5th Edition of the WHO 2022 Classification of Renal Neoplasms, there are over 20 subtypes of kidney tumors. However, RCCs are categorized into four main subtypes: clear cell tumors, papillary tumors, oncocytic and chromophobe tumors, and collecting duct tumors [22].

Patients with clear cell carcinoma, papillary type II, or variant histologies have the highest risk of recurrence. In contrast, chromophobe and papillary renal cell carcinomas generally have a better prognosis regarding recurrence risk [12]. Table 1 illustrates the global five-year recurrence risk stratified by tumor histology.

**Figure 2:**
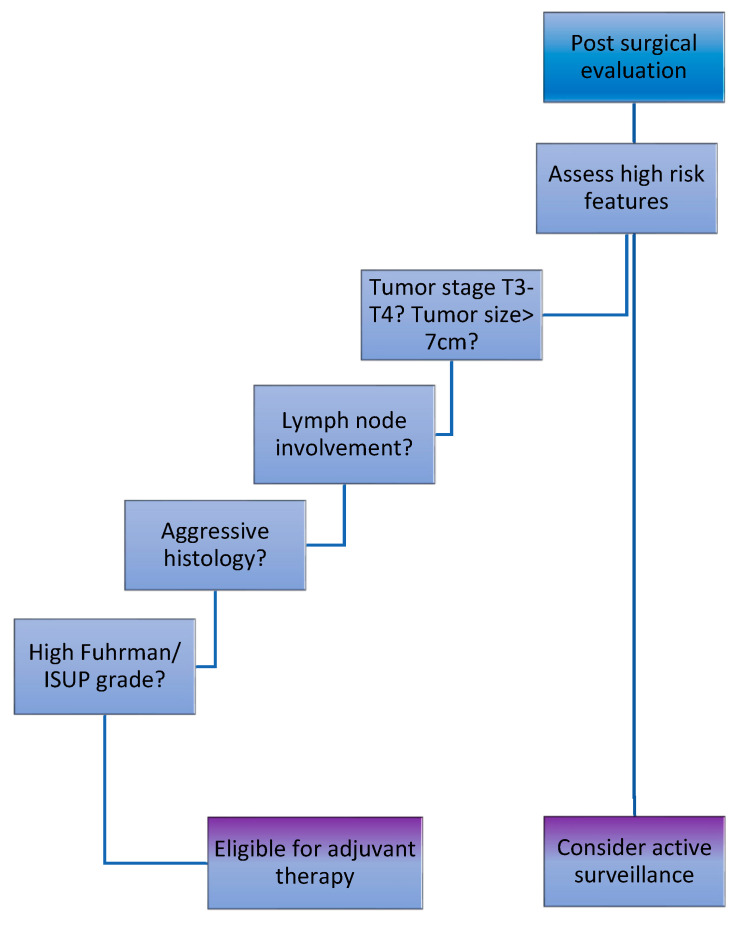
Important high-risk considerations for selecting adjuvant therapy.

In summary, adjuvant therapy is crucial for patients with localized RCC who face a significant risk of recurrence or metastasis after the complete surgical removal of their tumor. Key high-risk factors include histological subtype, tumor stage, size and grade, lymph node involvement, and positive surgical margins (Figure 2). Addressing these concerns with adjuvant therapy can enhance the chances of long-term survival and better outcomes for these patients.

## 3. How Can We Effectively Identify Patients Who Will Benefit Significantly from Adjuvant Therapy?

**Figure 3:**
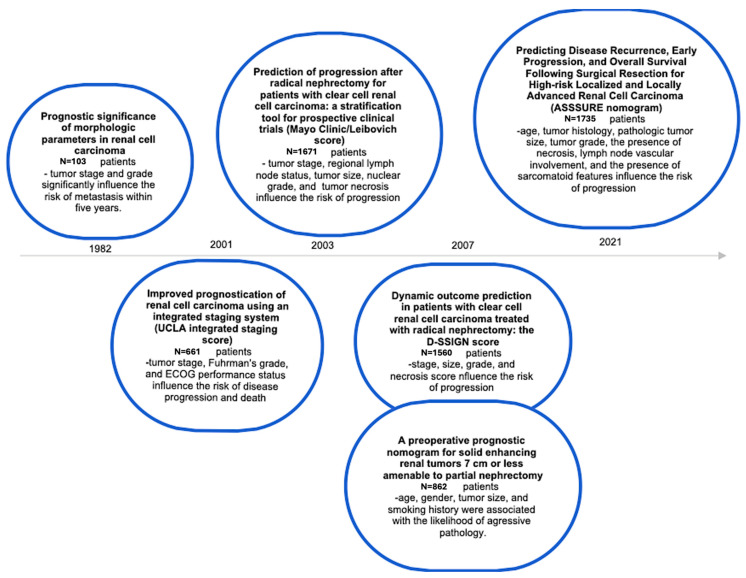
The chronological progression of prognostic indicators and nomograms for RCC.

Over the years, various scoring systems and nomograms have been employed to determine which patients with RCC are at an elevated risk of recurrence. Since the early 2000s, risk score stratification for RCC patients has enabled a more accurate selection of adjuvant therapies. Here is a clearer summary of the studies on risk stratification and prediction models for RCC outcomes (Figure 3).

In the early 1970s, Fuhrman and his colleagues identified tumor stage and grade as significant prognostic factors in RCC. They found that in Stage I RCC, tumors with lower grades (Grade 1) rarely metastasized within five years, whereas higher-grade tumors had a 50% metastasis rate. These data demonstrated that tumor grade is the most crucial factor in determining patient prognosis [26].

Later, in the 2000s, two models were implemented to predict cancer-specific survival rates after nephrectomy in localized RCC: the UCLA Integrated Staging Score and the Mayo Clinic/Leibovich Score. The UCLA algorithm combines clinical variables, such as TNM stage, Fuhrman’s grade, and ECOG performance status, and stratifies patients into five survival groups, with survival rates varying significantly from 96% at two years for the lowest-risk group to 0% for the highest-risk group [27]. The Mayo Clinic/Leibovich Score includes factors like age, tumor stage, lymph node status, and tumor size, all of which are significantly linked to the risk of metastasis or progression. These factors were also used for patient stratification in clinical trials [28,29].

With over 25 years of follow-up, the D-SSIGN score is another functional dynamic prediction tool for patients with localized RCC treated with radical nephrectomy. Based on tumor stage, size, grade, and necrosis, this score assesses tumor aggressiveness and predicts cancer-specific survival rates [30].

More recently, in 2021, Correa and his colleagues analyzed and enhanced the existing predictive models. The researchers used the ECOG-ACRIN 2805 (ASSURE) phase III RCC trial data to create a comprehensive postoperative recurrence prediction model. They analyzed 1735 patients with localized RCC who had undergone surgery and identified eight risk variables: age, tumor histology, size and grade, the presence of coagulative necrosis or sarcomatoid features, and lymph node or vascular involvement. Their study of these clinicopathological characteristics highlighted that tumor histology was the most powerful predictor of recurrence [31].

In conclusion, clinical and pathological features have remained valuable risk stratification models for RCC over the past five decades, offering valuable insights to improve patient care and influence surveillance and treatment decisions.

## 4. What Evidence Is Available Regarding Adjuvant Therapy RCC?

**Figure 4:**
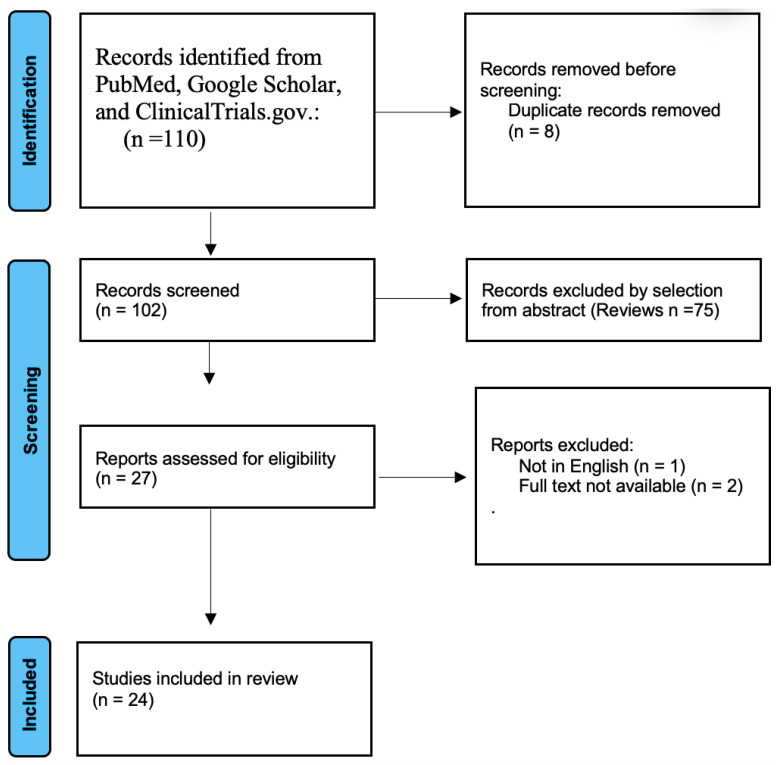
The PRISMA flow diagram used to identify clinical trials and assess their eligibility.

We conducted a systematic review of records regarding the use of various adjuvant regimens in RCC using databases such as PubMed, Google Scholar, and ClinicalTrials.gov, focusing exclusively on studies published entirely in English. Our search strategy included the following search terms in each database: “RCC” AND “adjuvant therapy”. We emphasized prospective studies and randomized trials up to January 2025 (Figure 4).

Following the database search, we identified 110 articles that met the main search criteria. After excluding records without full text, duplicates, and studies with incorrect designs, specifically reviews, we determined 24 articles to be eligible for inclusion. We identified 16 published clinical trials that explored adjuvant therapies for RCC and reported their results (Table 2). Additionally, we found eight emerging and ongoing trials related to this topic.

One of the earliest immunotherapies used in RCC is interferon alpha (IFN-*α*), along with high-dose interleukin-2 (IL-2). Their effectiveness has been strongly demonstrated in metastatic settings. However, clinical trials have also evaluated their efficacy in adjuvant settings [32,33,34,35].

In 2011, Aitchison et al. published findings from the EORTC/NCRI trial, which examined the effects of adjuvant treatments, specifically IL-2, IFN-*α*, and 5-fluorouracil, on patients at an elevated risk of relapse after undergoing the surgical removal of RCC. While the patients experienced significant side effects from these treatments, the study concluded that there was no improvement in disease-free or overall survival for the recipients of the adjuvant therapy [36].

Additional clinical trials investigated IFN-*α* as an adjuvant therapy following complete surgical resection, comparing it to observation alone. However, none of these trials demonstrated a statistically significant improvement in outcomes such as time to relapse or overall survival [37,38,39].

Other researchers have investigated the combination of granulocyte-macrophage colony-stimulating factor (GM-CSF) with IFN-*α* and IL-2 to effectively stimulate immune cells and promote anti-tumor immunity in RCC patients. In this context, Tsimafeyeu and his colleagues conducted a prospective, non-randomized trial to evaluate the toxicity and efficacy of a regimen of low-dose GM-CSF, IFN-*α*, and IL-2 administered postoperatively to patients with high-risk RCC. Although the trial revealed that the low-grade toxicities were manageable and the regimen was well-tolerated, the results indicated that this combination of low-dose GM-CSF, IFN-*α*, and IL-2 did not improve disease-free survival (DFS), compared to historical controls in patients with surgically resected high-risk RCC [40].

Although the direct stimulation of T cells with IFN-*α* or IL-2 has not demonstrated a benefit in the adjuvant setting, passive immunization using monoclonal antibodies has also been explored. Girentuximab specifically targets carbonic anhydrase IX (CAIX), a tumor-associated antigen widely expressed in clear cell RCC. Its safety and effectiveness were evaluated in the ARISER Randomized Clinical Trial [41]. Additionally, a vaccine derived from autologous tumors, known as HSPPC-96 (vitespen), was studied as an adjuvant treatment for patients at a high risk of recurrence following the resection of locally advanced RCC in a multicenter, open-label, randomized phase III trial [42]. In both trials, the researchers found no survival benefit in terms of DFS or overall survival.

Tyrosine kinase inhibitors (TKIs) have led to significant improvements in progression-free survival (PFS) and OS for patients with advanced RCC [43,44,45]. The ASSURE trial, a double-blind, placebo-controlled, randomized phase III study, evaluated the efficacy of TKIs, specifically sunitinib and sorafenib, in the adjuvant setting for high-grade, fully resected, non-metastatic RCC. The results revealed no significant differences in DFS between the TKI and placebo groups. The median DFS was 5.8 years for sunitinib (HR of 1.02), 6.1 years for sorafenib (HR of 0.97), and 6.6 years for the placebo. These findings indicated that adjuvants sunitinib and sorafenib did not provide a meaningful benefit. Additionally, the substantial toxicity associated with TKIs raised concerns about their tolerability in this context [46].

Sorafenib was also ineffective in the SORCE phase III trial, which demonstrated no significant improvement in DFS or OS, regardless of adjuvant therapy duration [47].

In contrast, the S-TRAC phase III trial demonstrated that adjuvant sunitinib significantly improved DFS in patients with high-risk, resected clear cell RCC, compared to the placebo. The median DFS in the sunitinib group was 6.8 years, compared to 5.6 years in the placebo group, with an HR of 0.73. Unfortunately, toxic effects were associated with this regimen, requiring frequent dose adjustments. However, this trial highlighted the potential of sunitinib as a feasible adjuvant therapy option for high-risk RCC [48].

What accounts for the differing results of the ASSURE trial (negative) and the S-TRAC trial (positive) in treating RCC? Key differences in trial design, patient populations, and methodologies played a critical role in these outcomes (Table 3).

The ASSURE trial did not demonstrate a benefit due to its diverse patient population, inclusion of lower-risk patients, and high rates of treatment discontinuation caused by treatment toxicity. In contrast, the S-TRAC trial showed a benefit by focusing on the high-risk clear cell RCC population, ensuring better adherence to therapy, and employing a more favorable trial design. These differences highlighted the importance of patient selection and trial methodology in evaluating adjuvant therapies for RCC.

Pazopanib and axitinib are two TKIs known for their efficacy in treating metastatic RCC. However, they have not demonstrated any benefits in adjuvant settings. The ATLAS trial was a phase III, randomized, double-blind study that evaluated the efficacy of adjuvant axitinib compared to a placebo in 724 patients with locoregional RCC at a high risk of recurrence after undergoing nephrectomy. The patients included in the study had tumors that invaded the muscle layer of the bladder wall, classified as ≥pT2 and/or N+ and were treated for up to three years or until disease recurrence or occurrence of unacceptable toxicity. The results indicated that adjuvant axitinib did not significantly improve DFS for patients with high-risk RCC following nephrectomy [49].

The PROTECT trial was another phase III, randomized trial that evaluated the efficacy of adjuvant pazopanib in patients with high-risk, locally advanced RCC after nephrectomy. One thousand five hundred thirty-eight patients were randomly assigned to receive either pazopanib (initially 800 mg daily, later adjusted to 600 mg for tolerability) or a placebo. Although the PROTECT trial did not show an overall DFS benefit for adjuvant pazopanib at the adjusted dose of 600 mg, a significant DFS improvement was observed in patients able to tolerate the full 800 mg dose (HR: 0.69, 95% CI: 0.51–0.94, P = 0.02). However, this trial did not provide an OS benefit, and due to its low treatment tolerability, it highlighted the challenges of balancing efficacy and toxicity in the adjuvant treatment setting [50].

As mentioned, RCC has been treated with immunotherapy for decades. Traditionally, immunotherapy has been used for advanced or metastatic RCC [51,52,53,54]. However, recent studies have shown that adjuvant immunotherapy, administered after surgery, can prevent recurrence [55,56,57,58,59,60,61].

The IMmotion010, CheckMate 914, KEYNOTE-564, and PROSPER trials evaluated different adjuvant immunotherapy therapies, specifically pembrolizumab, atezolizumab, nivolumab, and ipilimumab, in patients with RCC at a high risk of recurrence following nephrectomy. All the studies focused on high-risk patients but varied in their eligibility criteria [58,59,60,61]. Among these trials, KEYNOTE-564, which used adjuvant pembrolizumab, demonstrated a significant improvement in DFS for high-risk RCC patients after nephrectomy [60]. In contrast, the other trials, IMmotion010 (atezolizumab), CheckMate 914 (nivolumab plus ipilimumab), and PROSPER (perioperative nivolumab), did not indicate similar benefits [58,59,61]. Specifically, in the KEYNOTE-564 trial, the 24-month DFS rate was 77.3% for patients receiving pembrolizumab, compared to 68.1% for those receiving a placebo (HR 0.68 (0.53–0.87), *p* = 0.001). This positive outcome led to the approval of pembrolizumab for use in the adjuvant setting [60]. The PROSPER trial was unique, as it administered nivolumab both before and after surgery, while the other trials provided adjuvant therapy exclusively after surgery. However, this trial did not demonstrate a significant improvement in event-free survival when nivolumab was added to surgery, compared to surgery alone [61].

Numerous studies have highlighted the role of the mTOR signaling pathway in RCC growth [62,63,64]. Everolimus, a derivative of rapamycin that inhibits the mammalian target of rapamycin (mTOR) threonine kinase, has been approved as a second- and third-line therapy for patients with advanced RCC [65,66,67]. The EVEREST trial was a phase III, randomized, double-blind study that assessed the efficacy of adjuvant everolimus in patients with RCC at an intermediate, high, or extremely high risk of recurrence after undergoing nephrectomy. The trial found that everolimus did not significantly improve recurrence-free survival in the population studied [68].

Currently, several clinical trials are investigating adjuvant therapies for RCC (Table 4). These involve various combinations of immunotherapies, including durvalumab plus tremelimumab, belzutifan plus pembrolizumab, and toripalimab plus axitinib. Notably, the efficacy of an investigational mRNA-based personalized cancer vaccine, known as V940, in combination with pembrolizumab, is also being studied in high-risk RCC patients. Additionally, girentuximab, a chimeric monoclonal antibody specifically targeting CAIX, is under investigation. CAIX is a protein that is highly expressed in clear cell RCC but not found in normal kidney tissues. It serves as a hallmark of hypoxic tumor environments, making it an appealing target for therapies. Girentuximab is being evaluated for its potential synergistic effects when used alongside immune checkpoint inhibitors [69,70,71,72,73,74,75]. All the trials mentioned can be found on ClinicalTrials.gov and aim to assess the safety and efficacy of these combination therapies in preventing RCC recurrence after surgery. We look forward to their results.

## 5. Do Individual Patient Factors Play a Key Role in Choosing Adjuvant Therapy?

**Figure 5:**
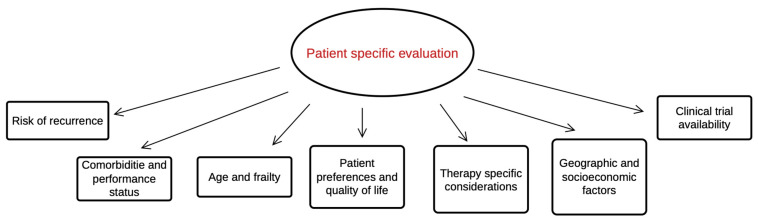
Individual considerations in selecting adjuvant therapy.

One of the most important aspects of adjuvant therapy is selecting the appropriate patients. Identifying which individuals with localized RCC are at a high risk for recurrence is crucial. We have identified various tumor-associated factors linked to recurrence and metastasis. However, clinical, biological, and personal risk factors should also be considered to ensure a highly individualized treatment approach (Figure 5) [76,77,78].

Recent findings have indicated that pembrolizumab is the only valid option for the adjuvant treatment of RCC [79]. An important factor to consider in immunotherapy is the possibility of adverse reactions. Immune-related adverse events (irAEs) occur due to increased and uncontrolled immune activation, which can trigger autoimmune responses affecting any organ or tissue. The most common immune toxicities involve the skin, endocrine organs, and the gastrointestinal system. However, more severe toxicities can affect the lungs, liver, kidneys, or heart, which can be challenging to identify as they often mimic symptoms of common illnesses or infections. Early recognition and management of these treatment-emergent adverse events are essential, typically using corticosteroids and other immunomodulatory agents in accordance with treatment guidelines [80,81].

Therefore, there are several absolute contraindications for immunotherapy, including severe, uncontrolled autoimmune diseases, a history of severe irAEs, and patients who are organ transplant recipients, such as those with kidney or liver transplants [82,83,84,85].

On the other hand, there are also some relative contraindications to immunotherapy, such as stable autoimmune diseases, which may be at risk of flare-ups [86,87,88]. Additionally, conditions including human immunodeficiency virus infections (HIV), hepatitis B and C, or tuberculosis necessitate an assessment of the patient’s infection status and management before the commencement of immunotherapy, as individuals with poorly controlled or active infections may face a higher risk of complications [89,90,91].

Patients with pre-existing interstitial lung disease or severe pulmonary conditions are at an elevated risk of experiencing immune-related pneumonitis, a serious and potentially fatal side effect of immunotherapy [92,93,94,95]. Further, neurological disorders, such as myasthenia gravis, Guillain–Barré syndrome, or peripheral neuropathy, may worsen with immune activation [96,97,98]. Medical history and comorbidity data are significant, especially for patients with a record of severe or unstable cardiovascular conditions at risk for immune-related myocarditis [99,100]. Additional attention should be paid to elderly and frail patients. While advanced age alone is not a contraindication for adjuvant treatment, factors such as frailty, comorbidities, and functional status should be considered [101,102,103,104].

Finally, the coadministration of multiple drugs can cause drug–drug interactions. Therefore, patients’ personal medications should be evaluated and checked for potential interactions with immunotherapy. For example, corticosteroids or immunosuppressive drugs for autoimmune diseases might compromise the effectiveness of immune checkpoint inhibitors in an adjuvant setting [105,106,107,108]. Table 5 presents a comprehensive overview of the common contraindications for immunotherapy, highlighting essential considerations for safe and effective treatment.

Importantly, there are socioeconomic differences in the accessibility of adjuvant therapies for RCC. For instance, access to advanced medical facilities, such as some immunotherapies or clinical trials, might be limited in developing countries due to unavailability and financial burdens. Therefore, improving equitable access to healthcare services requires collaboration among healthcare providers and enhanced access to knowledge and resources [109,110,111,112].

## 6. Conclusions and Feature Directions

Research increasingly focuses on identifying biomarkers that might play a relevant role in predicting which patients will benefit most from specific adjuvant therapies in RCC [113]. There is a strong correlation between RCC recurrence, treatment response, and a broad series of tissue biomarkers. For example, a recent study showed that a high expression of fibroblast activation protein-α was associated with the development of early RCC recurrence [114]. In addition, studies have reported that a high expression of TYROBP, a protein closely related to immune cell infiltration, including PD-L1 expression and CD8+ T cell infiltration, is associated with a low survival rate in clear cell RCC and a reduced treatment response to adjuvant immunotherapy [115]. Interestingly, even though immune checkpoint inhibitors have shown high efficacy in advanced renal cell carcinoma, high PD-1 and PD-L1 expressions have been associated with adverse RCC features such as larger renal tumor size or the presence of sarcomatoid features and, consequently, with poor outcomes [116].

Therefore, personalized medicine aims to improve treatment outcomes while reducing unnecessary side effects. Additionally, the latest advances in molecular genetics have greatly contributed to better risk stratification tools. These tools will help identify patients at a higher risk of recurrence who may benefit the most from more aggressive adjuvant therapy. A notable example is the use of whole-exome sequencing on resected tumors, including RCC, to enhance the selection of adjuvant treatments. In this context, Pastel and colleagues analyzed data from The Cancer Genome Atlas and validated the utility of a 16-gene recurrence score using RNA sequencing data. This transcriptomic recurrence score, based on a 16-gene assay, was associated with disease-free status and demonstrated a strong correlation with tumor recurrence [117]. Additionally, an experimental study assessed the somatic mutation status of 12 genes in 943 cases of clear cell RCC from a multinational patient cohort. The findings revealed that patients within the VHL-defined genomic group had significantly better outcomes compared to those whose tumors harbored VHL mutations along with additional alterations [118].

Furthermore, artificial intelligence and Machine Learning also have the potential to revolutionize adjuvant therapy selection in RCC by refining risk stratification, predicting treatment response, and enabling personalized therapeutic approaches. For example, artificial intelligence can analyze vast clinical, genomic, and imaging datasets to identify high-risk RCC patients who would benefit the most from adjuvant therapy [119,120].

Notably, ongoing studies are exploring many combination strategies, focusing on checkpoint inhibitors, targeted agents, and CAR-T cell therapy. These efforts aim to enhance efficacy, reduce toxicity, and achieve long-lasting remission [70,71,72,73,74,75]. Consequently, we await mature data from these trials, with extended follow-ups, to confirm the durability of responses and survival benefits.

In conclusion, is adjuvant therapy necessary for all patients with localized RCC? We still do not have a definitive answer to this question. The guidelines are comprehensive and subject to interpretation. Generally, the higher the tumor stage, the greater the risk of cancer recurrence. Consequently, patients with stage III and stage IV RCC who have undergone complete resection are at a high risk of recurrence, making the benefits of adjuvant therapy clear in these cases. However, for patients at a low risk of recurrence, such as those with stage II RCC, it is essential to identify high-risk features that may warrant consideration for adjuvant therapy. These features may include aggressive histological subtypes, tumor grade, or the presence of sarcomatoid differentiation.

It is also important to consider the potential for severe adverse reactions from immunotherapy, which could lead to treatment discontinuation or increased patient morbidity. Patient complexity presents significant challenges, as individuals with multiple chronic conditions require more attention and coordinated team efforts. Therefore, it is crucial to adopt a benefit–risk analysis approach, since quality of life is the most important measure of therapeutic benefit in cancer treatment.

## Figures and Tables

**Table 1:** Recurrence risk at five years based on tumor histology [12,23,24,25].

Histological Subtype	Recurrence Risk at 5 Years
Clear Cell RCC	10–70% (outcomes vary based on tumor stage, grade, and lymphovascular invasion)
Papillary RCC Type 1	A lower risk of recurrence
Type 2	20–40%
Chromophobe RCC	5–10%
Collecting Duct Carcinoma	50% within the first few years after surgery

**Table 2:** Summary of key highlights from critical trials and their impact on patient care.

Trial	Population Studied: High-Risk RCC	Histology	N	Drugs Studied	Primary End Point	Outcome	Adverse Events, Grade 3 or Higher	Discontinuations Due to Adverse Events	Interpretation
ASSURE	≥T1b	Clear cell and non-clear cell	1943	Sunitinib or sorafenib vs. placebo	DFS	HR 0.94 for sunitinib vs. placebo. HR 0.90; 97.5% CI, 0.71–1.14 for sorafenib vs. placebo	66% (sunitinib) 72% (sorafenib), 28% (placebo)	44% (sunitinib) 45% (sorafenib)	Sunitinib and sorafenib did not show any significant differences in DFS
S-TRAC	≥T3 and/or N+	Clear cell RCC	615	Sunitinib vs. placebo	DFS	HR 0.76; (0.59–0.98) *p* = 0.03	63.4% (sunitinib), 21.7% (placebo)	46.4% (sunitinib),	Sunitinib demonstrated a benefit to the use of TKIs (DFS benefit of 6.8 vs. 5.6 years)
PROTECT	pT2 (high grade) or ≥pT3	Clear cell RCC	1539	Pazopanib 800 mg, or pazopanib 600 mg vs. placebo.	DFS	HR 0.862 (0.699, 1.063); for or pazopanib 600 mg HR 0.693 (0.510, 0.943) for pazopanib 800	61% (pazopanib 800 mg), 48% (pazopanib 600 mg)	39% (pazopanib 800 mg), 35% (pazopanib 600 mg)	significant advantage in DFS for pazopanib 800 mg
SORCE	Leibovich score of 3 to 11	Clear cell and non-clear cell	1711	1 year of sorafenib, or 3 years of sorafenib vs. placebo	DFS	HR 1.01 (0.83–1.23) *p*= 0.95.	37%	24%	Sorafenib had no benefit in an adjuvant setting
ATLAS	≥pT2 and/or N+	Clear cell and non-clear cell	724	Axitinib vs. placebo	DFS	HR 0.870 (0.660–1.147) *p*= 0.3211	50%	17%	Axitinib did not improve DFS
EVEREST	pT1b G3–4, pT2–4, or pN+.	Clear cell and non-clear cell	11,545	Everolimus vs. placebo	rFS	HR 0.85 (0.72–1.00) *p* = 0.051	28%	20%	Everolimus did not improve rFS
KEYNOTE-564	pT2 G4 and/or sarcomatoid feature, pT3–4, or N+	Clear cell	994	Pembrolizumab vs. placebo		HR 0.72 (0.59–0.87 HR 0.62 (0.44–0.87) *p* = 0.005	19%	8%	Pembrolizumab demonstrated improvements in DFS and OS
IMmotion010	pT2 G4 N0, T3a G3–4 N0, T3b-c or T4 N0, N+	Clear cell	778	Atezolizumab vs. placebo	DFS	HR 0.93 (0.75–1.15), *p* = 0.50	16%	12%	Atezolizumab did not improve clinical outcomes
CheckMate 914	pT2a G3/4 N0, T2b-T4 any G N0, or N+	Clear cell	816	Part A: nivolumab + ipilimumab vs. placebo. Part B: nivolumab monotherapy vs. nivolumab + ipilimumab vs. placebo.	DFS	HR 0.92 (0.71–1.19) *p* = 0.53 for part A; HR 0.80 (0.58–1.12) *p* = 0.1936, for part B	16%	6% (nivolumab) 13% (nivolumab + ipilimumab)	No benefits were observed with the combination of adjuvant nivolumab and ipilimumab, nor with nivolumab alone.
PROSPER	T2 or greater, N+, oligoM1	Clear cell and non-clear cell	819	Perioperative nivolumab before nephrectomy followed by adjuvant nivolumab vs. surgery	EFS (event free survival)	HR 0.94(0.74–1.21) *p* = 0.32	48% (nivolumab+ surgery)	12%	Nivolumab did not improve rFS
ARISER	pT1b-T2 N0 G3–4, or >pT3N0M0 or N+	Clear cell RCC	864	Girentuximab vs. placebo	DFS OS	HR 0.97 (0.79–1.18) HR 0.99 (74–1.32)	13%	1%	Girentuximab demonstrated no clinical benefit
EORTC (GU Group)/NCRI trial	>T3b, or pN1–2, or microscopic positive margins, or microscopic vascular invasion	Clear cell and non-clear cell RCC	309	Aldesleukin (IL2)+ recombinant interferon alfa+ fluorouracil vs. observation	DFS OS	HR 0.84 (0.63–1.12) HR 0.86 (0.60–1.22)	48%	30–40%	No statistically significant benefit for the regimen in terms of DFS or OS
Granulocyte-Macrophage Colony-Stimulating Factor, Interferon Alpha and Interleukin-2 as Adjuvant Treatment for High-Risk Renal Cell Carcinoma	T3b-T4, or N1-N3, or M1 or T3a and G3	Clear cell and non-clear cell RCC	35	GM-CSF+ IFN-*α* + IL-2 vs. observation	DFS	DFS = 14.1 months (95% CI, 3–20.2 months) mOS = 53.0 months (95% CI, 40.6–65.4 months) *p* = 0.2	30–50%		No statistically significant improvement in DFS in comparison with historical control
Phase III study of interferon alfa-NL as adjuvant treatment for resectable renal cell carcinoma: an Eastern Cooperative Oncology Group/Intergroup trial	pT3–4a and/or N+	Clear cell and non-clear cell RCC	283	Observation vs. interferon alfa-NL	OS rFS	OS = 7.4 vs. 5.1, *p* = 0.9 rFS = 3.0 *v* 2.2 years, was not significantly longer (*p* = 0.33)	30–40%	30–40%	Interferon did not contribute to survival or rFS
Cytokine Working Group trial	T3b-4 or N1–3, or M1 with metastasectomy	Clear cell and non-clear cell RCC	69	IL-2 vs. observation	DFS	19.5 vs. 36 months, *p* = 0.43	30–40%	30–40%	IL-2 did not produce clinically meaningful benefit
A Multicenter, Randomized Phase III Study of Adjuvant Oncophage Versus Observation in Subjects With High Risk of Recurrence After Surgical Treatment for Renal Cell Carcinoma	cT1b–T4 N0 M0, or cTany N1–2 M0	Clear cell and non-clear cell RCC	818	An adjuvant autologous therapeutic vaccine (HSPPC-96; vitespen) vs. observation	rFS	HR 0.923, (0.729–1.169) *p* = 0.506	<10%	< 10%	The adjuvant autologous therapy did not lead to an improvement in rFS

Carbonic anhydrase IX = CAIX, disease free survival = DFS, granulocyte-macrophage colony stimulating factor = GM-CSF, no evidence of disease = NED, overall survival = OS, progression free survival = PFS, relapse free survival = rFS.

**Table 3:** Detailed breakdown between ASSURE and S-TRAC trials [46,48].

	ASSURE Trial	S-TRAC Trial
Histology	Both clear cell and non-clear cell RCC.	Exclusively clear cell RCC.
Risk stratification	Low-risk (pT1b or higher), intermediate-risk, and high-risk categories.	Only high-risk patients (T3 or higher, node-positive, or poor performance status).
Treatment duration and adherence	Significant toxicity led to higher rates of treatment discontinuation.	One year, potentially optimizing the exposure to sunitinib and its therapeutic effects. Managed to maintain adherence to the treatment regimen more effectively.
Trial endpoints	DFS was not stratified based on high-risk groups or clear cell RCC alone, leading to no significant differences between the arms.	DFS was a primary endpoint and was statistically significant in favor of sunitinib (HR 0.73, *p* = 0.03). The focus on a high-risk population allowed for a more robust demonstration of efficacy.
Statistical power and analysis	A larger group of patients (1943 patients) with wider inclusion criteria may have less statistical power to detect benefits in diverse populations.	A smaller, more focused group of 615 patients with strict criteria may have enhanced the observed treatment effect.
Toxicity management	The trial combined two different TKIs (sunitinib and sorafenib) with distinct toxicity profiles, complicating interpretation. High toxicity rates reduced tolerability and potentially affected outcomes.	Focused solely on sunitinib, and toxicity management was more streamlined, likely leading to better treatment adherence.

DFS = disease-free survival, HR = hazard ratio.

**Table 4:** Current clinical trials focusing on adjuvant therapy for RCC.

Trial	Disease Characteristics	Histology	Experimental Arms	Primary Outcome	Observations
An International Investigator-led Phase III Multi-Arm Multi-Stage Multi-center Randomized Controlled Platform Trial of Adjuvant Therapy in Patients With Resected Primary Renal Cell Carcinoma (RCC) at High or Intermediate Risk of Relapse (RAMPART).	Leibovich score of 3 to 11	All cell types of RCC are eligible, except for pure oncocytoma, collecting duct, medullary, and transitional cell cancer	Active monitoring, vs. durvalumab, vs. durvalumab plus tremelimumab	DFS OS	Trial recruiting
A Multicenter, Double-Blind, Randomized Phase 3 Study to Compare the Efficacy and Safety of Belzutifan (MK-6482) Plus Pembrolizumab (MK-3475) Versus Placebo Plus Pembrolizumab in the Adjuvant Treatment of Clear Cell Renal Cell Carcinoma (ccRCC) Post Nephrectomy (MK-6482-022).	pT2 G4, or >pT3 or/and N+, or M1 NED RCC with solid, isolated, soft tissue metastases that can be completely resected	Clear cell RCC	Belzutifan + pembrolizumab vs. placebo + pembrolizumab	DFS	Active trail, not recruiting
A Phase 2, Randomized, Double-Blind, Clinical Study of V940 (mRNA-4157) Plus Pembrolizumab (MK-3475) Versus Placebo Plus Pembrolizumab in the Adjuvant Treatment of Participants With Renal Cell Carcinoma (INTerpath-004).	pT2 G4, or >pT3 or/and N+, or M1 NED RCC with solid, isolated, soft tissue metastases that can be completely resected	Clear cell or papillary RCC	V940 + pembrolizumab vs. placebo + pembrolizumab	DFS	Recruiting
A Single-Arm Study of Toripalimab in Adjuvant Therapy After Resection of High-Risk Renal Cancer Tumors.	Stage III/IV	Clear cell and non-clear cell RCC	Toripalimab until tumor recurrence or intolerable toxicities	PFS OS	Recruiting
A Prospective, Multicenter, Single-Arm Clinical Study of the Efficacy and Safety of Toripalimab in Combination With Axitinib for Postoperative Adjuvant Therapy for Non-Clear Renal Cell Carcinoma With High-Risk Recurrence Factors	Papillary RCC, pT ≥ T1b and ISUP/WHO ≥ 3, N (any), M0; collecting duct carcinoma, SMARCB1-deficient renal medullary carcinoma, fumarate hydratase deficiency RCC (FH-RCC), any pT, any N M0.	Non-clear RCC except clear cell RCC, chromophobe RCC and eosinophilic RCC	Toripalimab + axitinib vs. monitoring	R	Recruiting
A Randomized, Double-Blind Phase III Study To Evaluate Adjuvant cG250 Treatment Versus Placebo In Patients With Clear Cell RCC And High Risk of Recurrence (ARISER).	T1b-T2 N0 M0, G 3–4 T3–T4 N0 M0 Any T N + M0	Clear cell RCC	Monoclonal chimeric antibody cG250 (girentuximab) vs. placebo	DFS OS	Trial completed
A Prospective, Multicenter, Single-arm Clinical Study of the Efficacy and Safety of Toripalimab in Combination With Axitinib for Postoperative Adjuvant Therapy for Non-Clear Renal Cell Carcinoma With High-Risk Recurrence Factors	pT ≥ T1b and ISUP/WHO ≥ 3, N (any), M0	Papillary Collecting duct carcinoma, SMARCB1-deficient renal medullary carcinoma, fumarate hydratase deficiency RCC	Toripalimab in combination with axitinib vs. placebo	DFS	Recruiting

**Table 5:** Cancer immunotherapy in special challenging populations [82,83,84,85,86,87,88,89,90,91,92,93,94,95,96,97,98,99,100,101,102,103,104,105,106,107,108].

Autoimmune Disorders There are over 100 autoimmune diseases, each with its own unique challenges and impacts, including well-known disorders like lupus, celiac disease, inflammatory bowel disease, type 1 diabetes, psoriasis, or neurological autoimmune diseases.	Considerations
	Immunotherapy may overstimulate the immune system, exacerbating autoimmune conditions. With close monitoring, some cases could still be eligible for treatment.
Organ transplantation	Immunotherapy can cause organ rejection by activating T cells. There may be exceptions depending on a risk–benefit assessment.
The use of high doses of corticosteroids (daily doses ≥ 10 mg of prednisone) or immunosuppressants such as methotrexate	Corticosteroids may compromise the efficacy of immunotherapy in the adjuvant setting by suppressing immune activation. Recommendations for glucocorticoid tapering.
History of anaphylaxis or severe allergic reactions	There is a risk of life-threatening hypersensitivity reactions, including cytokine release syndrome. Pre-treatment with antihistamines or steroids may be considered.
Bacterial, fungal and viral active infections	Immunotherapy can exacerbate infections Patients with well-managed infections may still qualify.
Pregnancy	Checkpoint inhibitors can cross the placenta and are associated with an increased incidence of pregnancy complications and prematurity.
Pre-existing cardiovascular (pericarditis, stroke, arrhythmias, or congestive heart failure) or lung abnormalities (pulmonary fibrosis)	These conditions have a high risk of morbidity and mortality and are linked to increased incidences of adverse events. Vigilant monitoring is essential.
